# In-hospital mortality risk stratification in children aged under 5 years with pneumonia with or without pulse oximetry: A secondary analysis of the Pneumonia REsearch Partnership to Assess WHO REcommendations (PREPARE) dataset

**DOI:** 10.1016/j.ijid.2023.02.005

**Published:** 2023-04

**Authors:** Shubhada Hooli, Carina King, Eric D. McCollum, Tim Colbourn, Norman Lufesi, Charles Mwansambo, Christopher J. Gregory, Somsak Thamthitiwat, Clare Cutland, Shabir Ahmed Madhi, Marta C. Nunes, Bradford D. Gessner, Tabish Hazir, Joseph L. Mathew, Emmanuel Addo-Yobo, Noel Chisaka, Mumtaz Hassan, Patricia L. Hibberd, Prakash Jeena, Juan M. Lozano, William B. MacLeod, Archana Patel, Donald M. Thea, Ngoc Tuong Vy Nguyen, Syed MA. Zaman, Raul O. Ruvinsky, Marilla Lucero, Cissy B. Kartasasmita, Claudia Turner, Rai Asghar, Salem Banajeh, Imran Iqbal, Irene Maulen-Radovan, Greta Mino-Leon, Samir K. Saha, Mathuram Santosham, Sunit Singhi, Shally Awasthi, Ashish Bavdekar, Monidarin Chou, Pagbajabyn Nymadawa, Jean-William Pape, Glaucia Paranhos-Baccala, Valentina Sanchez Picot, Mala Rakoto-Andrianarivelo, Vanessa Rouzier, Graciela Russomando, Mariam Sylla, Philippe Vanhems, Jianwei Wang, Sudha Basnet, Tor A. Strand, Mark I. Neuman, Luis Martinez Arroyo, Marcela Echavarria, Shinjini Bhatnagar, Nitya Wadhwa, Rakesh Lodha, Satinder Aneja, Angela Gentile, Mandeep Chadha, Siddhivinayak Hirve, Kerry-Ann F. O'Grady, Alexey W. Clara, Chris A. Rees, Harry Campbell, Harish Nair, Jennifer Falconer, Linda J. Williams, Margaret Horne, Shamim A. Qazi, Yasir Bin Nisar, Pneumonia REsearch Partnership to Assess WHO REcommendations (PREPARE) study group

**Affiliations:** a Division of Pediatric Emergency Medicine, Texas Children's Hospital/Baylor College of Medicine, Houston, United States of America; b Department of Global Public Health, Karolinska Institutet, Stockholm, Sweden and Institute for Global Health, University College London, London, United Kingdom; c Global Program in Respiratory Sciences, Eudowood Division of Pediatric Respiratory Sciences, Department of Pediatrics, Johns Hopkins School of Medicine, Baltimore, United States of America and Department of International Health, Johns Hopkins Bloomberg School of Public Health, Baltimore, United States of America; d Institute for Global Health, University College London, London, United Kingdom; e Ministry of Health, Lilongwe, Malawi; f Division of Vector—Borne Diseases, US Centers for Disease Control and Prevention, Fort Collins, United States of America; g Division of Global Health Protection, Thailand Ministry of Public Health–US Centers for Disease Control and Prevention Collaboration, Nonthaburi, Thailand; h South African Medical Research Council: Vaccines and Infectious Diseases Analytics Research Unit, School of Pathology, Faculty of Health Sciences, University of the Witwatersrand, Johannesburg, South Africa; Department of Science and Technology/National Research Foundation: Vaccine Preventable Diseases Unit, University of the Witwatersrand, Johannesburg, South Africa; i Pfizer Vaccines, Collegeville, United States of America; j The Children's Hospital, (Retired), Pakistan Institute of Medical Sciences (PIMS), Islamabad, Pakistan (deceased); k Advanced Pediatrics Centre, Postgraduate Institute of Medical Education and Research, Chandigarh, India; l Kwame Nkrumah University of Science & Technology/Komfo Anokye Teaching Hospital, Kumasi, Ghana; m World Bank, Washington DC, United States of America; n The Children's Hospital, Pakistan Institute of Medical Sciences (PIMS), Islamabad, Pakistan (deceased); o Department of Global Health, Boston University School of Public Health, Boston, United States of America; p University of KwaZulu-Natal, Durban, South Africa; q Florida International University, Miami, United States of America; r Lata Medical Research Foundation, Nagpur and Datta Meghe Institute of Medical Sciences, Sawangi, India; s Children Hospital No 1, Ho Chi Minh City, Vietnam; t Liverpool School of Tropical Medicine, Liverpool, United Kingdom; u Dirección de Control de Enfermedades Inmunoprevenibles, Ministerio de Salud de la Nación, Buenos Aires, Argentina; v Research Institute for Tropical Medicine, Manila, Philippines; w Department of Child Health, Faculty of Medicine, Universitas Padjadjaran, Bandung, Indonesia; x Shoklo Malaria Research Unit, Mae Sot, Thailand; y Rawalpindi Medical College, Rawalpindi, Pakistan; z Sana'a University, Sana'a, Yemen; aa Combined Military Hospital Institute of Medical Sciences, Multan, Pakistan; ab Instituto Nacional de Pediatria Division de Investigacion Insurgentes, Mexico City, Mexico; ac Children's Hospital Dr Francisco de Ycaza Bustamante, Head of Department, Infectious diseases, Guayaquil, Ecuador; ad Child Health Research Foundation and Dhaka Shishu Hospital, Dhaka, Bangladesh; ae International Vaccine Access Center (IVAC), Department of International Health, Johns Hopkins University, Baltimore, United States of America; af Medanta, The Medicity, Gurgaon, India; ag King George's Medical University, Department of Pediatrics, Lucknow, India; ah KEM Hospital Pune, Department of Pediatrics, Pune, India; ai University of Health Sciences, Rodolph Mérieux Laboratory & Ministry of Environment, Phom Phen, Cambodia; aj Mongolian Academy of Sciences, Academy of Medical Sciences, Ulaanbaatar, Mongolia; ak GHESKIO, Centre GHESKIO, Port au Prince, Haiti; al Fondation Merieux, Lyon, France; am Centre d'Infectiologie Charles Mérieux, Antananarivo, Madagascar; an GHESKIO, Department of Pediatrics, Port au Prince, Haiti; ao Universidad Nacional de Asuncion, Departamento de Biología Molecular y Genética, Instituto de Investigaciones en Ciencias de la Salud, Asuncion, Paraguay; ap Gabriel Touré Hospital, Department of Pediatrics, Bamako, Mali; aq Unité d'Hygiène, Epidémiologie, Infectiovigilance et Prévention, Hospices Civils de Lyon, Lyon, France and Centre International de Recherche en Infectiologie, Institut National de la Santé et de la Recherche Médicale U1111, CNRS Unité Mixte de Recherche 5308, École Nationale Supérieure de Lyon, Université Claude Bernard Lyon 1, Lyon, France; ar Chinese Academy of Medical Sciences & Peking Union, Medical College Institute of Pathogen Biology, MOH Key Laboratory of Systems Biology of Pathogens and Dr Christophe Mérieux Laboratory, Beijing, China; as Center for Intervention Science in Maternal and Child Health, University of Bergen, Norway and Department of Pediatrics, Tribhuvan University Institute of Medicine, Nepal; at Research Department, Innlandet Hospital Trust, Lillehammer, Norway; au Division of Emergency Medicine, Boston Children's Hospital, Harvard Medical School, Boston, United States of America; av PNUD/National University, Montevideo, Uruguay; aw Clinical Virology Unit, Centro de Educación Médica e Investigaciones Clínicas, Mar del Plata, Argentina; ax Translational Health Science and Technology Institute, Faridabad, India; ay All India Institute of Medical Sciences, New Delhi, India; az School of Medical Sciences & Research, Sharda University, Greater Noida, India; ba Department of Epidemiology, "R. Gutiérrez" Children's Hospital, Buenos Aires, Argentina; bb Former Scientist G, ICMR National Institute of Virology, Pune, India; bc KEM Hospital Research Center, Pune, India; bd Australian Centre for Health Services Innovation, Queensland University of Technology, Kelvin Grove, Australia; be Centers for Disease Control, Central American Region, Guatemala City, Guatemala; bf Division of Pediatric Emergency Medicine, Emory University School of Medicine, Children's Healthcare of Atlanta, Atlanta, United States of America; bg Centre for Global Health, Usher Institute, The University of Edinburgh, Edinburgh, Scotland; bh Department of Maternal, Newborn, Child, and Adolescent Health (Retired), World Health Organization, Geneva, Switzerland; bi Department of Maternal, Newborn, Child, and Adolescent Health and Ageing, World Health Organization, Geneva, Switzerland; bj African Leadership in Vaccinology Expertise (Alive), Faculty of Health Sciences, University of the Witwatersrand, Johannesburg, South Africa

**Keywords:** Pneumonia, Child, Danger sign, Chest indrawing, Under 5, Pulse oximetry

## Abstract

**Objectives:**

We determined the pulse oximetry benefit in pediatric pneumonia mortality risk stratification and chest-indrawing pneumonia in-hospital mortality risk factors.

**Methods:**

We report the characteristics and in-hospital pneumonia-related mortality of children aged 2-59 months who were included in the Pneumonia Research Partnership to Assess WHO Recommendations dataset. We developed multivariable logistic regression models of chest-indrawing pneumonia to identify mortality risk factors.

**Results:**

Among 285,839 children, 164,244 (57.5%) from hospital-based studies were included. Pneumonia case fatality risk (CFR) without pulse oximetry measurement was higher than with measurement (5.8%, 95% confidence interval [CI] 5.6-5.9% vs 2.1%, 95% CI 1.9-2.4%). One in five children with chest-indrawing pneumonia was hypoxemic (19.7%, 95% CI 19.0-20.4%), and the hypoxemic CFR was 10.3% (95% CI 9.1-11.5%). Other mortality risk factors were younger age (either 2-5 months [adjusted odds ratio (aOR) 9.94, 95% CI 6.67-14.84] or 6-11 months [aOR 2.67, 95% CI 1.71-4.16]), moderate malnutrition (aOR 2.41, 95% CI 1.87-3.09), and female sex (aOR 1.82, 95% CI 1.43-2.32).

**Conclusion:**

Children with a pulse oximetry measurement had a lower CFR. Many children hospitalized with chest-indrawing pneumonia were hypoxemic and one in 10 died. Young age and moderate malnutrition were risk factors for in-hospital chest-indrawing pneumonia-related mortality. Pulse oximetry should be integrated in pneumonia hospital care for children under 5 years.

## Highlights

• Children with pneumonia whose oxygen level was measured had a lower risk of death.
• Hypoxemia was frequent among danger signs and chest-indrawing pneumonia cases.
• Pulse oximeters are essential tools for hospital-based child pneumonia care.
• Additional interventions to reduce in-hospital pneumonia deaths should be explored.

## Background

Pneumonia and other acute lower respiratory infections (ALRIs) remain the leading cause of death in children aged 1-59 months [1]. Over the last 2 decades, substantial progress has been made to reduce mortality and limit unnecessary hospitalizations. Randomized controlled trials demonstrated that children aged 2-59 months with chest-indrawing pneumonia without any general danger sign experience similar treatment failure rates with oral amoxicillin as those managed with injectable penicillin [2], [3], [4]. In response to these findings, in 2012 the World Health Organization (WHO) revised their pneumonia management guideline [5], which was included in the second edition of the WHO Pocket book of hospital care for children [6] and the Integrated Management of Childhood Illness chart booklet [7] in 2014 (Box 1) [6,8]. It recommends that children aged 2-59 months without HIV with chest indrawing but without general danger signs (unable to drink/feed; convulsions; sleepy/lethargic; vomiting everything; severe wheezing; and signs of respiratory distress, including grunting, head nodding, nasal flaring), stridor, severe malnutrition, or hypoxemia (defined as a peripheral transcutaneous oxyhemoglobin saturation [SpO_2_] <90%) can be treated with oral amoxicillin. Other trials in India [9], Malawi [10], Kenya [11], and Pakistan [12,13] and two observational studies in Papua New Guinea [14] and Kenya [15] demonstrated that these children could be safely treated with oral antibiotics at home. However, most studies screened for and excluded hypoxemic children, using definitions ranging from SpO_2_ <90% to <85%. In addition, none were powered to demonstrate the differences in mortality [4].

Box 1 **2005 versus 2013 WHO pneumonia hospitalization criteria for those aged 2-59 months.**

**Table:** 

Pneumonia classification	WHO pocketbook 2005	WHO pocketbook 2013
Non-severe (outpatient treatment)	Fast breathing^a^	Fast breathing^a^ and/or chest indrawing
Severe (hospitalize)	Chest indrawing	General danger sign^b^ or oxygen saturation <90%
Very severe (hospitalize)	General danger signs^b^	Not applicable

a Fast breathing for age: RR ≥50 bpm in those aged 2-11 months and RR ≥40 bpm in those aged 12-59 month;

b Danger signs are either according to WHO pocketbook (i.e., central cyanosis, apnea, gasping, grunting, nasal flaring, severe wheezing, head nodding) or according to IMCI general danger sign (inability to drink, lethargy or unconscious, convulsions, vomit everything), stridor in a calm child or weight-for-age z-score <-3. bpm, breaths per minute; RR, respiration rate WHO, World Health Organization.

Alt-text: Unlabelled box

Chest indrawing is a cardinal feature of respiratory distress that precedes hypoxemia and respiratory failure in children. This inward movement of abdominal and chest wall soft tissue below the rib cage is due to the increased negative intrapleural pressures generated to expand lungs with poor compliance during inspiration [16]. Hypoxemia occurs most commonly when there is ventilation perfusion mismatch in the lungs from an ALRI and is most frequently measured noninvasively by a pulse oximeter device [17]. However, before COVID-19, when the data for the current analysis were collected, pulse oximetry was limited in many low-resource settings, particularly in primary and community care [18,19]. It is recognized that in the absence of pulse oximetry, the WHO Integrated Management of Childhood Illness protocol may miss hypoxemia, leading to misclassification of patients who need oxygen and inpatient care [20], [21], [22], [23], [24], [25]. A retrospective Kenyan study conducted in district hospitals without pulse oximeters found that, apart from danger signs, mild to moderate pallor, age <12 months, lower chest indrawing, respiratory rate of 70 breaths or more, admission to a hospital in a malaria-endemic region, and moderate malnutrition were independently associated with pneumonia-related mortality [26].

The overall goal of this study was to understand the value of pulse oximetry in evaluating hospitalized children with pneumonia. We also explored additional clinical characteristics that were risk factors for chest-indrawing pneumonia mortality and could therefore be used to identify children with a high mortality risk. Using the WHO Pneumonia Research Partnership to Assess WHO Recommendations (PREPARE) study dataset, we aimed to (i) describe and compare the clinical characteristics and case fatality risk (CFR) by pneumonia severity among children with and without a pulse oximetry reading at study enrollment and (ii) determine in-hospital mortality risk factors among children aged 2-59 months with chest-indrawing pneumonia, with and without pulse oximetry measurements.

## Methods

### Study sample

**Figure 1:**
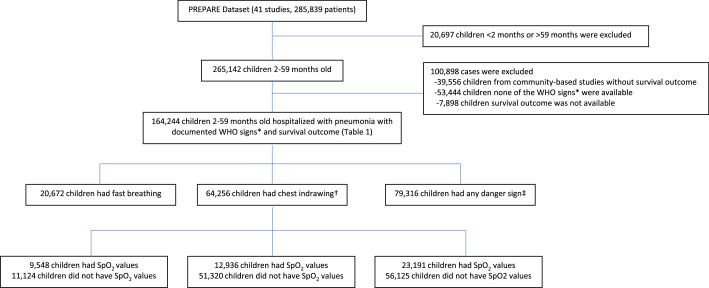
WHO signs: Fast breathing (respiratory rate 50 or more per minute in 2-11 months old and 40 or more per minute in 12-59 months old), lower chest indrawing, or danger signs (defined below)†Of 26 hospital-based studies in the PREPARE dataset, 17 studies reported information on the presence of chest indrawing in children 2-59 months of age.‡Danger signs are either according to WHO pocketbook (i.e., central cyanosis, apnea, gasping, grunting, nasal flaring, severe wheezing, head nodding) or according to IMCI general danger sign (inability to drink, lethargy or unconscious, convulsions, vomit everything), stridor in a calm child or weight-for-age z-score <-3. SpO_2_: oxygen saturation; WHO, World Health Organization.

We conducted a secondary analysis of collated datasets from 41 studies included in the WHO PREPARE project. These studies were conducted in 31 countries, including 29 low-middle-income countries (LMICs). Figure 1 describes how the analytic dataset was selected and used for this analysis. A detailed description of the studies is shown in Table 1 [2,^3^,[27], [28], [29], [30], [31], [32], [33], [34], [35], [36], [37], [38], [39], [40], [41], [42], [43], [44], [45], [46], [47], [48]. The primary data collection occurred between 1994 and 2014, and countries were at varying stages of Pneumococcus and *Haemophilus influenzae* type B (Hib) vaccine implementation.

**Table 1:** Characteristics of hospital-based studies included in the analysis (n = 164,244).

First author	Study design	Years of study	Country(ies) of study	Year of introduction		N	SpO_2_ 93-100%, n (%)	SpO_2_ 90-92%, n (%)	SpO_2_<90%, n (%)	No SpO_2_ value, n (%)	Chest indrawing, n (%)	Deaths, n (%)
				PCV	HiB							
Addo-Yobo	Randomized controlled trial	1998-2000	Colombia Ghana India Mexico Pakistan South Africa Vietnam Zambia	2011 2012 No 2009 2014 2009 No 2013	1998 2002 2015 1999 2009 1999 2010 2004	1628	1041 (63.9%)	323 (19.8%)	240 (14.7%)	24 (1.5%)	1534 (94.2%)	15 (0.9%)
Ugpo	Prospective observational	1994-2000	Philippines	2020	2012	1097	678 (61.8%)	227 (20.7%)	190 (17.3%)	2 (0.2%)	436 (39.7%)	19 (1.7%)
Basnet	Randomized controlled trial	2006-2008	Nepal	2015	2009	638	68 (10.7%)	146 (22.9%)	423 (66.3%)	1 (0.2%)	192 (30.1%)	6 (0.9%)
Mathew	Prospective cohort	2011-2013	India	No	2015	1833	1192 (65.0%)	283 (15.4%)	341 (18.6%)	17 (0.9%)	0 (0.0%)	148 (8.2%)
Clara	Retrospective cohort	2011-2013	Panama	2010	2000	46	34 (73.9%)	1 (2.2%)	9 (19.6%)	2 (4.3%)	11 (23.9%)	1 (2.4%)
Marcone	Prospective, cross-sectional	2008-2010	Argentina	2012	1997	497	41 (8.2%)	76 (15.3%)	32 (6.4%)	348 (70.0%)	0 (0.0%)	0 (0.0%)
Benet	Prospective, case-control study	2010-2014	Cambodia China Haiti India Madagascar Mali Paraguay	2015 No 2018 No 2012 2011 2012	2010 No 2012 2015 2008 2007 2002	833	519 (62.3%)	150 (18.0%)	79 (9.5%)	85 (10.2%)	215 (25.8%)	19 (2.3%)
McCollum	Prospective cohort	2012-2014	Malawi	2011	2002	14,032	9629 (68.6%)	1190 (8.5%)	1352 (9.6%)	1861 (13.3%)	2658 (18.9%)	439 (3.1%)
Lazzerini	Prospective cohort	2001-2012	Malawi	2011	2002	101,182	0 (0.0%)	0 (0.0%)	0 (0.0%)	101,182 (100.0%)	46,160 (45.6%)	6027 (6.0%)
Gentile	Retrospective observational	2001-2013	Argentina	2012	1997	305	64 (21.0%)	111 (36.4%)	115 (37.7%)	15 (4.9%)	0 (0.0%)	3 (1.0%)
Gessner	Retrospective cohort	1999-2001	Indonesia	No	2014	5244	2177 (41.5%)	1238 (23.6%)	1780 (33.9%)	49 (0.9%)	4174 (79.6%)	483 (9.2%)
Lu	Retrospective cross-sectional with follow-up	2005-2010	Thailand	No	2019	18,942	6664 (35.2%)	977 (5.2%)	791 (4.2%)	10,510 (55.5%)	3777 (19.9%)	106 (0.6%)
Hazir	Randomized controlled trial	2005-2007	Pakistan	2014	2009	2067	0 (0.0%)	0 (0.0%)	0 (0.0%)	2067 (100%)	1916 (92.7%)	0 (0.0%)
Hirve	Prospective observational	2009-2011	India	No	2015	249	0 (0.0%)	0 (0.0%)	0 (0.0%)	249 (100%)	0 (0.0%)	0 (0.0%)
Hortal	Prospective observational	2009-2012	Uruguay	2008	1994	553	413 (74.7%)	79 (14.3%)	56 (10.1%)	5 (0.9%)	401 (72.5%)	6 (1.1%)
Wulandari	Retrospective cohort	2012-2016	Indonesia	No	2014	1089	276 (25.3%)	200 (18.4%)	330 (30.3%)	283 (26.0%)	249 (22.9%)	61 (5.6%)
Klugman	Randomized controlled trail	1998-2000	South Africa	2009	1999	8113	4557 (56.2%)	1813 (22.4%)	1581 (19.5%)	162 (2.0%)	675 (8.3%)	418 (5.1%)
Neuman	Prospective cohort	2006-2009	USA	2000	1985	576	501 (87.0%)	41 (7.1%)	19 (3.3%)	15 (2.6%)	0 (0.0%)	0 (0.0%)
O'Grady	Randomized controlled trial	2001-2002	Australia	2001	1993	90	76 (84.4%)	6 (6.7%)	1 (1.1%)	7 (7.8%)	17 (18.9%)	0 (0.0%)
Ferrero	Prospective observational	1998-2002	Argentina	2012	1997	1,357	0 (0.0%)	0 (0.0%)	0 (0.0%)	1357 (100%)	1233 (90.9%)	21 (1.5%)
Asghar	Randomized controlled trial	2000-2004	Bangladesh Ecuador India Mexico Pakistan Yemen Zambia	2015 2010 No 2009 2014 2011 2013	2009 2003 2015 1999 2009 2005 2004	894	180 (20.1%)	131 (14.6%)	577 (64.5%)	6 (0.7%)	0 (0.0%)	46 (5.1%)
Turner	Prospective observational	2007-2008	Thailand	No	2019	952	602 (63.2%)	183 (19.2%)	37 (3.9%)	130 (13.7%)	276 (29.0%)	0 (0.0%)
Wadhwa	Randomized controlled trial	2007-2010	India	No	2015	438	350 (79.9%)	66 (15.1%)	17 (3.9%)	5 (1.1%)	256 (58.4%)	7 (1.6%)
Cutts	Randomized controlled trial	2002-2004	Gambia	2009	1997	1589	1179 (74.2%)	113 (7.1%)	110 (6.9%)	187 (11.8%)	76 (4.8%)	96 (6.0%)

PCV: Pneumoccocus conjugate vaccine, HiB: *Haemophilus influenzae* type B, SpO_2_: oxygen saturation

### Inclusion and exclusion criteria

The patient records of hospitalized children aged 2-59 months with WHO-defined pneumonia and had survival outcome were included in the current analyses. We excluded patient records from community-based studies, those without WHO signs for pneumonia classification, or those who had no survival outcomes (Figure 1). Children received hospital-based care, including antibiotics and supplemental oxygen when indicated and available according to local norms.

### Definitions and variables

The WHO pneumonia severity was defined as fast breathing (respiratory rate above the age-specific cut-off), chest indrawing, or danger sign (Box 1). Before the 2012 WHO guidance revision [5], it was recommended that all children with chest indrawing, even those without danger signs, should be hospitalized for injectable antibiotics and supportive care. Variables were chosen *a priori* due to clinical significance and potential association with mortality based on previous studies. These variables include: age, sex, weight, weight-for-age z-score, temperature (normothermia [35.5-37.9°C], fever [≥38°C], and hypothermia [<35.5°C]), age-adjusted tachypnea, severe tachypnea (defined as respiratory rate ≥70 breaths per minute), signs of severe respiratory distress (*i.e*., grunting, head nodding, or nasal flaring), and SpO_2_ (if reported) [6,^7^,20]. Although pallor and residence in malaria hyperendemic regions have been previously described as pneumonia-related mortality risk factors [26], these data were not routinely included in our dataset. Some of the studies that contributed to the dataset were multicountry and included malaria hyperendemic regions and nonhyperendemic regions, but the dataset identifies cases by study and does not include if the case was from a specific country. Our primary outcome of interest was in-hospital pneumonia-related mortality.

### Statistical analysis

To address the first objective, we described and compared the frequency, proportion with 95% confidence interval (CI), mean, median, and missingness of data on the clinical characteristics at the time of admission by pneumonia severity and if pulse oximetry was measured. Then, we reported the CFR by WHO pneumonia severity and compared these CFRs by the presence of pulse oximetry measurements. Pulse oximetry was categorized into no SpO_2_ measurement and any SpO_2_ measured at presentation. The measured group was further stratified into SpO_2_ <90%, 90-92%, and 93-100% to explore the impact of these categories on CFR [49,50].

For the second objective, we exclusively used the data from the chest-indrawing pneumonia cases subset to fit two mixed-effects logistic regression models to explore the associations between demographic characteristics, nutritional status, and clinical signs at initial presentation with mortality. Mixed-effects modeling was chosen because some parameters had clear fixed effects on the outcome (tachypnea vs no tachypnea), whereas we assumed other parameters had a variable or unknown effect on the outcome (male vs female). In addition, we included variables to reflect the study type (observational vs randomized controlled trial) and pneumococcal vaccine (PCV) implementation at time of data collection because these factors had clear effects on mortality. Heterogeneity was accounted for at each study level. The first model included chest-indrawing cases with pulse oximetry measurements at hospitalization, and the second included those without pulse oximetry measurements. In the model that included pulse oximetry, SpO_2_ categories (<90%, 90-92%, and >93%) were treated as ordinal co-variables. To assess for bias, we described the variable missingness. We then conducted a bivariate analysis with complete cases. Variables with >15% missingness were excluded from the multivariable model. We reported the adjusted odds ratio (aOR) with 95% CI.

## Results

### Included studies

Among the 285,839 children from 41 studies in the PREPARE dataset, 164,244 (57.5%) from 26 of the 41 included studies (conducted in 29 countries) met the inclusion criteria for analysis (Figure 1 and Table 1). All cases that met the inclusion criteria were enrolled in-hospital-based studies. Pulse oximetry measurements were reported in 27.8% (n = 45,675) of cases. Among the 164,244 children included in the analyses, there were 7921 deaths (CFR 4.8%). Of included cases, 12.6% (n = 20,672) had only fast-breathing pneumonia, 39.1% (n = 64,256) had chest indrawing with or without fast breathing, and 48.3% (n = 79,316) had any danger sign at the time of admission.

The majority of cases with a pulse oximetry measurement (62.5%, 28,554/45,675) were from three studies conducted in Malawi [33] (26.6%, n = 12,171), Thailand [37] (18.5%, n = 8432), and South Africa [41] (17.4%, n = 7951) (Table 1). For chest-indrawing pneumonia cases with pulse oximetry measurements (n = 12936), 20.5% were from Malawi [33] and 32.3% were from Indonesia [36].

A countrywide pneumonia surveillance study conducted in Malawi from 2001 to 2012 provided 85.3% (n = 101,182/118,569) of cases without pulse oximetry measurement [34]. This study contributed 90.0% (n = 46,160/51,320) of the cases with chest-indrawing pneumonia without pulse oximetry measurement. Four studies with overall CFRs ≥6.0% were from India [29] (n = 1833, CFR 8.2%), Indonesia [36] (n = 5244, CFR 9.2%), the Gambia [48] (n = 1589, CFR 6.0%), and Malawi [34] (n = 101,182, CFR 6.0%).

### Clinical characteristics

The overall hypoxemia (SpO_2_ <90%) prevalence was 17.7% (95% CI 17.3-18.0%). A nearly similar prevalence of hypoxemia was observed in patients with chest-indrawing pneumonia (19.7%; 95% CI 19.0-20.4%) and patients with danger sign pneumonia (20.7%; 95% CI 20.2-21.2%). Of the 164,244 cases in the dataset, 6.1% reported data on signs of severe respiratory distress. Children with chest-indrawing pneumonia with and without pulse oximetry measurements had similar characteristics except for differences in the prevalence of temperature ≥38°C (35.9% with vs 40.5% without SpO_2_) and severe tachypnea (10.7% with vs 13.9% without SpO_2_), which might reflect the differences in frequency of missing data among cases without an SpO_2_ measurement (Table 2). Among fast-breathing pneumonia cases, a larger proportion of children with *versus* without pulse oximetry measurements were aged 2-5 months (14.1% with vs 9.7% without SpO_2_). Otherwise, the demographic and clinical characteristics by pneumonia severity were similar between the SpO_2_ measured and not measured cohorts.

**Table 2:** Baseline characteristics of hospitalized children aged 2-59 months with pneumonia by pneumonia classification and pulse oximetry assessment (N = 164,244).

Baseline characteristics	Fast breathing (n = 20,672)		Chest indrawing (n = 64,256)		Danger signs^a^ (n = 79,316)	
	Pulse oximetry not measured (n = 11,124)	Pulse oximetry measured (n = 9548)	Pulse oximetry not measured (n = 51,320)	Pulse oximetry measured (n = 12,936)	Pulse oximetry not measured (n = 56,125)	Pulse oximetry measured (n = 23,191)
**Study design** Clinical trial Observational study	56 (0.5, 0.4, 0.6) 11,068 (99.5, 99.3, 99.6)	1259 (13.2, 12.5, 13.9) 8289 (86.8, 86.1, 87.5)	1949 (3.8, 3.6, 4.0) 49,371 (96.2, 96.0, 96.4)	2717 (21.0, 20.3, 21.7) 10,219 (79.0, 78.3, 79.7)	454 (0.8, 0.7, 0.9) 55,671 (99.2, 99.1, 99.3)	9022 (8.9, 38.3, 39.5) 14,169 (61.1, 60.5, 61.7)
**Pneumococcal vaccine rollout** Yes No	29 (0.3, 0.2, 0.4) 11,095 (99.7, 99.6, 99.8)	903 (9.5, 8.9, 10.1) 8645 (90.5, 89.9, 91.1)	546 (1.1, 1.0, 1.2) 50,774 (98.9, 98.8, 99.0)	2524 (19.5, 18.8, 20.2) 10,412 (80.5, 79.8, 81.2)	1293 (2.3, 2.2, 2.4) 54,832 (97.7, 97.6, 97.8)	9336 (40.3, 39.6, 40.9) 13,855 (59.7, 59.1, 60.4)
**Age in months** median value, (IQR) **Age category** 2-5 months old, n (%, 95% CI) 6-11 months old, n (%, 95% CI) 12-59 months old, n (%, 95% CI)	18.4 (12.2, 28.1) 1084 (9.7, 9.2-10.3) 1592 (14.3, 13.7-14.9) 8448 (75.9, 75.1-76.7)	17.6 (10.5, 27.1) 1345 (14.1, 13.4- 14.8) 1371 (14.4, 13.7-15.1) 6832 (71.6, 70.6- 72.5)	10.0 (5.0, 17.9) 17,636 (33.3, 32.9-33.7) 14,608 (28.5, 28.1-28.8) 19,636 (38.3, 37.8-38.7)	9.5 (5.0, 17.8) 4117 (31.8, 31.0-32.6) 3762 (29.1, 28.3-29.9) 5057 (39.1, 38.2-39.9)	10.0 (5.0, 19.0) 18,509 (33.0, 32.6-33.4) 15,259 (27.2, 26.8-27.6) 22,357 (39.8, 39.4-40.2)	10.1 (5.0, 18.9) 7088 (30.6, 30.0-31.2) 6379 (27.5, 26.9-28.1) 9724 (41.9, 41.3-42.6)
**Sex** Male, n (%, 95% CI) Female, n (%, 95% CI) Missing, n (%)	6102 (54.8, 53.9-55.8) 4944 (44.4, 43.5-45.4) 78 (0.7)	5466 (57.2, 56.2-58.2) 4049 (42.4, 41.4-43.4) 33 (0.4)	27,302 (53.2, 52.8-53.6) 22,939 (44.7, 44.3-45.1) 1,079 (2.1)	6591 (50.9, 50.1-51.8) 6290 (48.6, 47.7-49.4) 55 (0.4)	30,783 (54.8, 54.4-55.2) 24,263 (43.2, 42.8-43.6) 1079 (1.9)	13,342 (57.5, 56.9-58.1) 9689 (41.8, 41.1-42.4) 160 (0.7)
**Weight** (in kg) Mean (SD) Missing, n (%)	10.3 (3.1) 3604 (32.4)	10.2 (3.4) 1362 (14.3)	8.4 (2.7) 2171 (4.2)	8.4 (3.53) 840 (6.5)	8.1 (2.8) 1866 (3.3)	8.1 (3.1) 1356 (5.8)
**WAZ** >-2, n (%, 95% CI) -3 <WAZ ≤-2, n (%, 95% CI) <-3 WAZ Missing, n (%)	6373 (57.3, 56.4-58.2) 947 (8.5, 8.0-9.0) NA 3804 (34.2)	6760 (70.8, 69.9-71.7) 1123 (11.8, 11.1-12.4) NA 1665 (17.4)	39,279 (76.5, 76.2-76.9) 7357 (14.3, 14.0-14.6) NA 4684 (9.1)	9680 (74.8, 74.1-75.6) 1964 (15.2, 14.6-15.8) NA 1292 (10.0)	35,211 (62.7, 62.3-63.1) 6747 (12.0, 11.7-12.3) 9738 (17.3, 17.0-17.7) 4429 (7.9)	14,073 (60.7, 60.0-61.3) 2637 (11.4, 11.0-11.8) 4076 (17.6, 17.1-18.1) 2405 (10.4)
**Body temperature** 35.5-37.9 C, n (%, 95% CI) ≥38.0 C, n (%, 95% CI) <35.5 C, n (%, 95% CI) Missing, n (%)	4765 (42.8, 41.9-43.8) 6042 (54.3, 53.4-55.2) 20 (0.2, 0.1-0.3) 297 (2.7)	5350 (56.0, 55.0-57.0) 4036 (42.3, 41.3-43.3) 21 (0.2, 0.1-0.3) 141 (1.5)	22,936 (44.7, 44.3-45.1) 20,808 (40.5, 40.1-41.0) 364 (0.7, 0.-0.8) 7212 (14.0)	8147 (63.0, 62.1-63.8) 4641 (35.9, 35.0-36.7) 37 (0.3, 0.2-0.4) 111 (0.8)	24,501 (43.6, 43.2-44.1) 21,389 (38.1, 37.7-38.5) 662 (1.2, 1.1-1.3) 9573 (17.1)	14,588 (62.9, 62.3-63.5) 7777 (33.5, 32.9-34.1) 201 (0.9, 0.7-1.0) 625 (2.7)
**Respiratory rate** (breaths/min) median (IQR) **Respiratory rate category** ≥70, n (%, 95% CI) <70, n (%, 95% CI) Missing, n (%)	48.0 (42.0, 58.0) 623 (5.6, 5.2-6.0) 10,501 (94.4, 93.9-94.8) 0 (0.0)	50.0 (44.0,58.0) 416 (4.4, 3.9-4.8) 9132 (95.6, 95.2-96.0) 0 (0.0)	60.0 (53.0-66.0) 7136 (13.9, 13.6-14.2) 38,749 (75.5, 75.1-75.9) 5435 (10.6)	56.0 (48.0, 64.0) 1379 (10.7, 10.1-11.2) 11,034 (85.3, 84.7-85.9) 523 (4.0)	60.0 (54.0, 68.0) 10,315 (18.4, 18.1-18.7) 38,351 (68.3, 67.9-68.7) 7459 (13.3)	56.0 (48.0, 64.0) 3302 (14.2, 13.8-14.7) 19,293 (83.2, 82.7-83.7) 596 (2.6)
**SpO_2_** Median value (%), (IQR) **SpO_2_ category** SpO_2_ 93-100%, n (%, 95% CI) SpO_2_ 90-92%, n (%, 95% CI) SpO_2_ < 90%, n (%, 95% CI)	NA	96.0 (93.0, 97.0) 7572 (79.3, 78.5-80.1) 1243 (13.0, 12.3-13.7) 733 (7.7, 7.1-8.3)	NA	94.0 (90.0, 96.0) 8015 (62.0, 61.1-62.8) 2375 (18.4, 17.7-19.0) 2546 (19.7, 19.0-20.4)	NA	94.0 (90.0, 97.0) 14,654 (63.2, 62.6-63.8) 3736 (16.1, 17.8-19.1) 4801 (20.7, 20.2-21.2)
**Outcome** Death, n (%, 95% CI) Survived, n (%, 95% CI)	97 (0.9, 0.7-1.1) 11,027 (99.1, 98.9-99.3)	61 (0.6, 0.5-0.8) 9487 (99.4, 99.2-99.5)	1496 (2.9, 2.8-3.1) 49,824 (97.1, 96.9-97.2)	450 (3.5, 3.2-3.8) 12,486 (96.5, 96.2-96.8)	4,747 (8.5, 8.2-8.7) 51,378 (91.5, 91.3-91.8)	1070 (4.6, 4.3-4.9) 22,121 (95.4, 95.1-95.6)

SpO_2_: peripheral capillary oxyhemoglobin saturation, IQR: interquartile range, CI: confidence interval, SD: standard deviation, WAZ: WHO weight-for-age z-score; WHO, World Health Organization.

a Danger signs are either according to WHO pocketbook [6]; i.e.; central cyanosis, apnea, gasping, grunting, nasal flaring, audible wheeze, head nodding) or according to IMCI [7]; i.e.; general danger sign (inability to drink, lethargy or unconscious, convulsions, vomit everything), stridor in a calm child or weight-for-age z-score <-3.

### Case fatality risk

In Table 3, we compare the CFR of cases with and without pulse oximetry integrated into their care. Column 1 reflects the data from four studies with 100% missing SpO_2_ values. Pulse oximetry was not documented in these studies because it was not integrated into the overall study design. Column 2 reflects the data from studies with <100% missing values. Except for two retrospective studies by Lu (55.5%) and Wulandari (26.0%), all studies had <15% missing SpO_2_ values. We chose to include the Lu and Wulandari studies in column 2 because the subanalyses suggested that pulse oximetry measurements were missing at random because there was no difference in the CFR among children with and without a documented SpO_2_ measurement. Most missing measurements were in children with fast-breathing pneumonia, and pulse oximetry is routinely not used in these cases.

**Table 3:** CFR of children aged 2-59 months hospitalized with pneumonia by pulse oximetry assessment and pneumonia classification (n = 164,244).

Parameters	Children in which SpO_2_ reading was not available		Children with any SpO_2_ reading	Children in which SpO_2_ reading was available		
				SpO_2_ reading category		
	Studies with 100% missing values	Studies with 1-99% missing values		SpO_2_ <90%	SpO_2_ 90-92%	SpO_2_ 93-100%
	Deaths/total (CFR, 95% CI)	Deaths/total (CFR, 95% CI)	Deaths/total (CFR, 95% CI)	Deaths/total (CFR, 95% CI)	Deaths/total (CFR, 95% CI)	Deaths/total (CFR, 95% CI)
**Any severity of pneumonia**	**6048/104855** **(5.8%, 5.6-5.9%)**	**292/13714** **(2.1%, 1.9-2.4%)**	**1581/45675** **(3.5%, 3.3-3.6%)**	**851/8080** **(10.5%, 9.9-11.2%)**	**186/7354** **(2.5%, 2.2-2.9%)**	**544/30241** **(1.8%, 1.6-1.9%)**
**Pneumonia Classification**						
Fast breathing	57/1944 (2.9%, 2.2-3.8%)	40/9180 (0.4%, 0.3-0.6%)	61/9548 (0.6%, 0.5-0.8%)	20/733 (2.5%, 1.7-4.2%)	7/1243 (0.6%, 0.2-1.1%)	34/7572 (0.4%, 0.3-0.6%)
Lower chest indrawing	1446/49309 (2.9%, 2.8-3.1%)	50/2011 (2.5%, 1.8-3.3%)	450/12936 (3.5%, 3.2-3.8%)	262/2546 (10.3%, 9.1-11.5%)	61/2375 (2.6%, 2.0-3.3%)	127/8015 (1.6%, 1.3-1.9%)
Danger signs^a^	4545/53602 (8.5%, 8.2-8.7%)	202/2523 (8.0%, 7.0-9.1%)	1070/23191 (4.6%, 4.3-4.9%)	569/4801 (11.8%, 10.9-12.8%)	118/3736 (3.2%, 2.6-3.8%)	383/14654 (2.6%, 2.4-2.9%)

CFR, case fatality risk; CI, confidence interval; SpO_2_, peripheral capillary oxyhemoglobin saturation.

a Danger signs are either according to World Health Organization pocketbook; i.e.; central cyanosis, apnea, gasping, grunting, nasal flaring, severe wheezing, head nodding) or according to IMCI; i.e.; general danger sign (inability to drink, lethargy or unconscious, convulsions, vomit everything), stridor in a calm child or weight-for-age z-score <-3.

The CFR of patients without pulse oximetry measurement integrated as part of their care was significantly higher than those with a recorded measurement (5.8% vs 2.1%) (Table 3). This was particularly notable in children with any danger sign because those without a pulse oximetry measurement had a CFR of 8.5% (95% CI 8.2-8.7%) *versus* 4.6% (95% CI 4.3-4.9%) among those with a measurement. In patients with SpO_2_ measurement, independent of hypoxemia, the overall CFR of chest-indrawing pneumonia was 3.5% (95% CI 3.2-3.8%) *versus* 4.6% (95% CI 4.3-4.9%) in danger sign cases. Hypoxemic pneumonia cases of SpO_2_ <90% with a danger sign or chest indrawing had high CFRs of 11.8% and 10.3%, respectively. The CFR of patients with chest indrawing with SpO_2_ <90% (10.3%, 9.1-11.5%) was four times higher than those with an SpO_2_ of 90-92% (2.6%; 95% CI 2.0-3.3%) and six times higher than those with an SpO_2_ of 93-100% (1.6%; 95% CI 1.3-1.9%) (Table 3).

### Mortality risk factors among chest-indrawing cases

We report the models of risks for chest-indrawing pneumonia-associated mortality with (Table 4) and without (Supplementary Tables 1 and 2) pulse oximetry measurements. Of cases with a measured SpO_2,_ age bands 2-5 months (aOR 9.94, 95% CI 6.67-14.84) and 6-11 months (aOR 2.01, 95% CI 1.27-3.18), SpO_2_ <90% (aOR 3.47, 95% CI 2.66-4.52), -3 <weight-for-age z-score <-2 (aOR 2.67, 95% CI 1.71-4.16), and female sex (aOR 1.82, 95% CI 1.43-2.32) were associated with in-hospital mortality (Table 4). Notably, 41.8% (95% CI 37.2-46.5%; 188/450) of deaths occurred in children with an SpO_2_ >90%. Children with chest-indrawing pneumonia without a pulse oximetry measurement had similar pneumonia-related mortality risk factors (Supplementary Table 1). Because 90.0% of these cases came from Malawi, we conducted a sensitivity analysis by excluding Malawi cases and found that only moderate malnutrition remained a mortality risk factor (Supplementary Table 2).

**Table 4:** Clinical characteristics associated with death of children aged 2-59 months hospitalized with chest-indrawing pneumonia with pulse oximetry assessment (n = 12,936).

Variable	Bivariate				Adjusted OR^a^ (95% CI)
	Died, (n = 450) n (%)	Survived, (n = 12486) n (%)	OR (95% CI)	*P*-value	
**Study design** Clinical trial Observational study	43 (1.6) 407 (4.0)	2,674 (98.4) 9,812 (96.0)	0.39 (0.28-0.53) 1.00 (reference)	<0.0001	0.25 (0.16-0.37) 1.00 (reference)
**Pneumococcal vaccine rollout** Yes No	34 (1.4) 416 (4.0)	2,490 (98.6) 9,996 (96.0)	0.33 (0.23-0.47) 1.00 (reference)		0.36 (0.22-0.60)1.00 (reference)
**Age categories** 2-5 months 6-11 months 12-59 months	303 (7.4) 97 (2.6) 50 (1.0)	3,814 (92.6) 3,665 (97.4) 5,007 (99.0)	7.95 (5.88-10.76) 2.65 (1.88-3.74) 1.00 (reference)	<0.0001 <0.0001	9.94 (6.67-14.84) 2.67 (1.71-4.16) 1.00 (reference)
**Sex** Male Female Missing	170 (2.6) 278 (4.4) 2 (3.6)	6,421 (97.4) 6,012 (95.6) 53 (96.4)	1.00 (reference) 1.75 (1.44-2.12) ——–	<0.0001	1.00 (reference) 1.82 (1.43-2.32)
**WAZ categories** WAZ >-2 -3 <WAZ <-2 Missing	249 (2.6) 114 (5.8) 87 (6.7)	9,431 (97.4) 1,850 (94.2) 1,205 (93.3)	1.00 (reference) 2.33 (1.86-2.93) ——–	<0.0001	1.00 (reference) 2.41 (1.87-3.09)
**Body temperature** Normal temperature (35.5-37.9 C) Fever (≥38.0 C) Hypothermia (<35.5 C) Missing	291 (3.6) 150 (3.2) 2 (5.4) 7 (6.3)	7,856 (96.4) 4,491 (96.8) 35 (94.6) 104 (93.7)	1.00 (reference) 0.90 (0.74-1.10) 1.54 (0.37-6.44) ——–	0.311 0.552	1.00 (reference) 0.81 (0.64-1.03) 2.17 (0.28-17.02)
**Respiratory rate (breaths/min)** Respiratory rate <70 breaths/min Respiratory rate ≥70 breaths/min Missing	355 (3.2) 83 (6.0) 12 (2.3)	10,679 (96.8) 1,296 (94.0) 511 (97.7)	1.00 (reference) 1.93 (1.50-2.46) ——–	<0.0001	1.00 (reference) 1.31 (0.98-1.76)
**SpO_2_ categories** SpO_2_ 93-100% SpO_2_ 90-92% SpO_2_ <90%	127 (1.6) 61 (2.6) 262 (10.3)	7,888 (98.4) 2,314 (97.4) 2,284 (89.7)	1.00 (reference) 1.64 (1.20-2.23) 7.12 (5.74-8.85)	0.002 <0.0001	1.00 (reference) 1.36 (0.96-1.92) 4.14 (3.19-5.36)

OR: odds ratio; WAZ: weight-for-age z-score; SpO_2_: oxygen saturation.

a Adjusted for study design, PCV rollout, age, sex, weight-or-age z-score, body temperature, respiratory rate and oxygen saturation.

## Discussion

In this study, the CFR of cases with an SpO_2_ measurement was lower than those without. Hypoxemia of SpO_2_ <90% was highly prevalent among children with chest-indrawing or danger sign pneumonia. Patients with chest-indrawing and danger sign pneumonia with an SpO_2_ <90% had a CFR of 10.3% and 11.8%, respectively. Age bands 2-5 months and 6-11 months, SpO_2_ <90%, moderate malnutrition, and female sex were independently associated with chest-indrawing pneumonia-related in-hospital death. We used a large multicountry dataset of hospitalized patients with pneumonia to explore the clinical outcomes in child pneumonia cases with and without SpO_2_ measurement and focused on cases with chest-indrawing pneumonia. Given the size of the dataset, our *post hoc* power estimate was greater than 95%. All data-contributing studies were conducted before the implementation of the 2012 WHO pneumonia management guidance recommending that children with chest-indrawing pneumonia without danger signs or hypoxemia (if pulse oximetry is available) could be safely managed in outpatient settings with oral amoxicillin [5].

The CFR was higher among child pneumonia cases without a documented SpO_2_ measurement. The reduced CFR with pulse oximeter use may reflect the impact of pulse oximetry on hospital outcomes or effects of a more functional health system [51]. Healthcare worker identification of hypoxemia likely influenced if a child received supplemental oxygen [52,53]. In contrast, clinicians may use pulse oximetry as an objective measurement to improve their assessments, which, in some cases, may result in the de-escalation of unnecessary care, presumably freeing up resources for children who could benefit from them [54]. Pulse oximetry implementation has been shown to increase the diagnosis of pneumonia [55], improve the overall quality of care for pneumonia and malaria [19,56], and decrease hospital-based pediatric ALRI mortality, independent of supplemental oxygen availability [57].

Our hypoxemia prevalence and some of our CFR findings differ from other published reports. Our estimates are slightly higher than that of a 2009 metanalysis [58]. This study included a small amount of data (<10%) from studies that enrolled children aged up to 12 years. Hypoxemic pneumonia is less frequent in older children, which may explain their slightly lower estimates. In contrast, our findings are much lower than that reported by two meta-analyses [59,60] and the Pneumonia Etiology Research for Child Health (PERCH) study [61] (47-35.8%). Misclassification bias could explain these differences. In the PERCH study, at most study sites, hypoxemia was defined as an SpO_2_ <92%. In Rahman *et al.*’s metanalysis, they were unable to disaggregate data, and 17 of the 57 included studies defined hypoxemia as an SpO_2_ <92-95% [60]. All four studies presented combined chest-indrawing and danger sign cases into one cohort when describing hypoxemia frequency. In previous works [49,^50^,62] conducted in countries with a high anemia prevalence [63], an SpO_2_ of 90-92% was proposed as an ALRI-associated mortality risk factor, independent of clinical severity, but this was not associated with chest-indrawing pneumonia mortality in our data. In our study, hypoxemia (SpO_2_ <90%) prevalence was high and it put children, particularly those with chest indrawing or danger signs, at risk for death. Despite the WHO's recommendation to use pulse oximetry to assess hypoxemia and clear evidence that pulse oximeters are essential medical devices, many outpatient facilities and hospitals in LMICs do not have or do not use pulse oximeters in routine care [18,^19^,[64], [65], [66], [67]. Unfortunately, clinical signs alone are not reliable predictors of hypoxemia, resulting in both false-positive and false-negative classifications, leading to many hypoxemic children not receiving oxygen and potentially contributing to pneumonia-related deaths [20,^53^,68]. Global uptake of pulse oximeters at the health system level will take time due to funding and implementation challenges, such as procurement, training, promotion of use, and ongoing monitoring and feedback [19,69]. Nonetheless, pulse oximetry implementation is a necessary investment.

The value of pulse oximetry is clear; however, other factors may play an important role in reducing chest-indrawing pneumonia deaths. Unlike children with chest-indrawing pneumonia receiving outpatient care, the children in this study had access to supplemental oxygen and hospital-based care and received injectable antibiotics yet they still died. Similar to ours, other studies identified age 2-11 months, moderate malnutrition, severe tachypnoea, and female sex as mortality risk factors among children with pneumonia [26,^49^,70]. These risk factors are plausible. Sex-based health disparities, including delayed care seeking, have been demonstrated in Africa and Asia and may explain some of these findings [26,^49^,[71], [72], [73]. The excess mortality burden in infancy may reflect incomplete vaccination or a higher risk of occult untreated serious bacterial infection other than pneumonia, such as bacteremia, urinary tract infection, malaria, and meningitis [74,75]. Exploratory studies are necessary to identify how these risk factors could inform medical decision making in the triage, follow-up, and hospital care of children with chest-indrawing pneumonia. Although known to contribute to pneumonia-related [76] and all-cause mortality, there is no formal disease-specific guidance on the care of children who are moderately malnourished. Targeted interventions could reduce pneumonia-related mortality in this group. For instance, the association of enteral protein intake during hospitalization with reduced 60-day mortality is well documented in critically ill children who are mechanically ventilated, independent of baseline nutrition status [77]. It is plausible that protein supplementation could reduce pneumonia-related mortality, particularly in children who are moderately malnourished. An ongoing phase II randomized controlled trial in Kenya and Uganda addresses this issue in children with severe (danger sign) pneumonia [78]. Other potential studies could evaluate if close outpatient follow-up, earlier hospital referral, or more intensive in-hospital monitoring of select groups, such as young infants aged 2-5 months, may reduce hospitalized pneumonia deaths.

### Limitations

This study had some limitations. First, most of these data are derived from studies conducted before or during the widespread PCV and Hib vaccine implementation. Accordingly, we may be overestimating the prevalence of severe and hypoxemic pneumonia and its associated mortality because 73% of infants now receive Hib vaccine, and around 45% receive PCV [79]. However, suggesting otherwise, our findings have a similar CFR as that of the PERCH study (6.7%), which examined the etiology of severe pneumonia in the postpneumococcal vaccination era [61]. Second, because the studies included in our dataset occurred before the WHO recommendation that chest-indrawing cases be managed with oral amoxicillin, the majority of these cases received injectable antibiotics. Third, HIV co-morbidity data were not commonly documented in the dataset. Pneumonia-associated hypoxemia and mortality are higher among children who are HIV-positive or -exposed [80]. Therefore, our findings are not generalizable to this high-risk patient group. In addition, other co-infections, such as malaria, were not accounted for [74]. Although, this may also reflect real-world conditions in settings without reliable HIV and malaria testing resources. Fourth, there are inherent differences in pulse oximeter devices, training, and supervision, which could affect the accuracy of SpO_2_ measurements and our findings. Fifth, only 6.1% of this dataset included information on signs of respiratory distress. Given that these are considered danger signs that warrant hospitalization, we cannot be certain that some of the cases with chest indrawing did not also have signs of respiratory distress. Sixth, we collated data from a diverse range of settings and a large proportion of unmeasured pulse oximetry cases came from a single 10-year study in Malawi. To address this, we conducted a sensitivity analysis and found that moderate malnutrition remained a mortality risk factor even when the Malawian cases were excluded. Seventh, we were unable to assess for study-level variance in the duration of illness before hospitalization, length of hospitalization, and time to inpatient death, which may reflect the differences in care-seeking behaviors and clinician judgment to hospitalize a patient. There are certainly other unmeasured factors affecting child pneumonia in-hospital mortality, which we are unable to account for. Finally, we aggregated data from a wide variety of studies conducted in many different countries, which may limit the applicability of our findings to local contexts. Some of the data are from clinical trials or prospective studies with dedicated study staff, whereas others are from routine care settings; as such, there is variability in the quality of the reported data. We are unable to account for how management differences affected the patient outcomes. From an implementation perspective, the use of data collected outside of a funded, well-staffed, and well-supplied clinical trial may be a strength because these data more accurately reflect the real-world conditions of healthcare delivery in LMICs.

## Conclusion

Pulse oximetry use is critical to providing effective pneumonia care. Given that many LMIC ALRI care settings do not have or use pulse oximeters and that danger signs and chest-indrawing cases had a high prevalence of hypoxemia and associated CFR, we can conclude that many children who could benefit from supplemental oxygen are going unrecognized. This represents a missed opportunity to reduce child pneumonia deaths. A substantial proportion of chest-indrawing pneumonia deaths were not hypoxemic. Exploratory research is needed to understand how mortality risk factors, such as moderate malnutrition and young age, could be used to guide care to reduce mortality. Our findings suggest that pulse oximetry should be integrated in the clinical evaluation of children aged 5 years who are hospitalized with ALRI, particularly for children with chest-indrawing pneumonia.

## Declaration of competing interest

The authors have no competing interests to declare. YBN is a staff member of the WHO. The expressed views and opinions do not necessarily represent the policies of the WHO.

## Funding

The Bill and Melinda Gates Foundation, Seattle, WA, USA (#INV-007927).

## Ethical approval

All studies included in this deidentified data set were previously granted clearance by ethical review boards at each participating site.

## Appendix Supplementary materials

Image, application 1

Image, application 2
