# Supplementary material for: In-hospital mortality risk stratification in children aged under 5 years with pneumonia with or without pulse oximetry: A secondary analysis of the Pneumonia REsearch Partnership to Assess WHO REcommendations (PREPARE) dataset
Source: Int J Infect Dis. 2023 Apr;129:240–50. doi: 10.1016/j.ijid.2023.02.005 (PMC10017350; doi:10.1016/j.ijid.2023.02.005)
Supplement: Supplementary file 1 [file mmc1.docx]

| **Supplementary Table 1. Clinical characteristics associated with death of 2-59 months old children hospitalized with chest indrawing pneumonia without pulse oximetry assessment (n=51,320)** | | | | | |
| --- | --- | --- | --- | --- | --- |
| Variable | Bivariate | | | | Adjusted* OR (95% CI) |
|  | Died, n (%) | Survived, n (%) | Odds Ratio  (95% CI) | p-value |  |
| **Study design**  Clinical trial  Observational study | 3 (0.2)  1493 (3.0) | 1946 (99.8)  47878 (97.0) | 0.05 (0.02-0.15)  1.00 (reference) | <0.0001 | 0.02 (0.01-0.15)  1.00 (reference) |
| **Pneumococcal vaccine (PCV) rollout**  Yes  No | 26 (4.8)  1470 (2.9) | 520 (95.2)  49304 (97.1) | 1.68 (1.13-2.49)  1.00 (reference) | 0.011 | 3.12 (1.12-8.74)  1.00 (reference) |
| **Age categories**  2-5 months  6-11 months  12-59 months | 696 (4·1)  447 (3·1)  353 (1·8) | 16,380 (95·9)  14,161 (96·9)  19,283 (98·2) | 2·32 (2·04-2·64)  1·72 (1·50-1·98)  1·00 (reference) | <0·0001  <0·0001 | 2·70 (2·27-3·20)  1·90 (1·58-2·29)  1·00 (reference) |
| **Sex**  Male  Female  Missing | 676 (2·5)  766 (3·3)  54 (5·0) | 26,626 (97·5)  22,173 (96·7)  1,025 (95·0) | 1·00 (reference)  1·36 (1·22-1·51)  -------- | <0·0001 | 1·00 (reference)  1·44 (1·26-1·64) |
| **Weight-for-age z-score (WAZ) categories**  WAZ > -2  -3 < WAZ < -2  Missing | 802 (2·0)  368 (5·0)  326 (7·0) | 38,477 (98·0)  6,989 (95·0)  4,358 (93·0) | 1·00 (reference)  2·53 (2·23-2·86)  -------- | <0·0001 | 1·00 (reference)  2·74 (2·38-3·16) |
| **Body temperature**  Between 35·5 and 37·9C  > 38·0 C  < 35·5 C  Missing | 639 (2·8)  554 (2·7)  32 (8·8)  271 (3·8) | 22,297 (97·2)  20,254 (97·3)  332 (91·2)  6,941 (96·2) | 1·00 (reference)  0·95 (0·85-1·07)  3·36 (2·32-4·87)  -------- | 0·428  <0·0001 | 1·00 (reference)  1·01 (0·88-1·16)  2·35 (1·42-3·90) |
| **Respiratory rate (breaths/min)**  Respiratory rate <70 breaths/min  Respiratory rate >70 breaths/min  Missing | 982 (2·5)  335 (4·7)  179 (3·3) | 37,767 (97·5)  6,801 (95·3)  5,256 (96·7) | 1·00 (reference)  1·89 (1·67-2·15)  -------- | <0·0001 | 1·00 (reference)  1·72 (1·47-2·00) |
|  |  |  |  |  |  |
| OR: Odd ratio; WAZ: Weight for age z-score;  *Adjusted for study design, PCV rollout, age, sex, weight-or-age z-score, body temperature, and respiratory rate. | | | | | |
