# Supplementary material for: In-hospital mortality risk stratification in children aged under 5 years with pneumonia with or without pulse oximetry: A secondary analysis of the Pneumonia REsearch Partnership to Assess WHO REcommendations (PREPARE) dataset
Source: Int J Infect Dis. 2023 Apr;129:240–50. doi: 10.1016/j.ijid.2023.02.005 (PMC10017350; doi:10.1016/j.ijid.2023.02.005)
Supplement: Supplementary file 2 [file mmc2.docx]

| **Supplementary Table 2. Clinical characteristics associated with death of 2-59 months old hospitalized children with chest indrawing pneumonia without pulse oximetry assessment (n=5,160), excluding the Malawi study** | | | | | |
| --- | --- | --- | --- | --- | --- |
| Variable | Bivariate | | | | Adjusted* OR (95% CI) |
|  | Died, n (%) | Survived, n (%) | Odds Ratio  (95% CI) | p-value |  |
| **Age categories**  2-5 months  6-11 months  12-59 months | 21 (1·5)  18 (1·3)  25 (1·0) | 1,357 (98·5)  1,345 (98·7)  2,394 (99·0) | 1·48 (0·83-2·66)  1·28 (0·70-2·36)  1·00 (reference) | 0·187  0·425 | 2·34 (0·63-8·70)  1·39 (0·36-5·35)  1·00 (reference) |
| **Sex**  Male  Female  Missing | 32 (1·1)  32 (1·5)  0 (0·0) | 2,927 (98·9)  2,121 (98·5)  48 (100·0) | 1·00 (reference)  1·38 (0·84-2·26)  -------- | 0·201 | 1·00 (reference)  1·78 (0·60-5·25) |
| **Weight-for-age z-score (WAZ) categories**  WAZ > -2  -3 < WAZ < -2  Missing | 19 (0·6)  9 (1·9)  36 (2·2) | 3,033 (99·4)  462 (98·1)  1,601 (97·8) | 1·00 (reference)  3·11 (1·40-6·91)  -------- | 0·005 | 1·00 (reference)  3·76 (1·21-11·71) |
| **Body temperature**  Between 35·5 and 37·9C  > 38·0 C  < 35·5 C  Missing | 20 (0·7)  31 (1·5)  2 (22·2)  11 (4·0) | 2,783 (99·3)  2,043 (98·5)  7 (77·8)  263 (96·0) | 1·00 (reference)  2·11 (1·20-3·71)  39·76 (7·78-203·29)  -------- | 0·010  <0·0001 | 1·00 (reference)  0·93 (0·29-2·98)  † |
| **Respiratory rate (breaths/min)**  Respiratory rate <70 breaths/min  Respiratory rate >70 breaths/min  Missing | 27 (0·8)  1 (0·4)  36 (2·1) | 3,181 (99·2)  225 (99·6)  1,690 (97·9) | 1·00 (reference)  0·52 (0·07-3·87)  -------- | 0·526 | 1·00 (reference)  † |
|  |  |  |  |  |  |
| OR: Odd ratio; WAZ: Weight for age z-score;  *Adjusted for study, age, sex, weight-or-age z-score, body temperature, and respiratory rate.  †Given small total sample size (N<10) or univariate p-value >0.20 was not calculated. | | | | | |
